# 

**Published:** 1877-11-20

**Authors:** 


					﻿VOL. VII.	NOVEMBER 20. 1877.	No. 11.
THE
SOUTHERN MEDICAL RECORD.
A MONTHLY JOURNAL OF PRACTICAL* MEDICINE.
“ QUJCQUID PR^CIPIES ESTO BREVIS. ”
i &
s
w
W
h
W
! A
i <i
! O
i HI
A
. «
P
O
f*
■	o
; a
i
i«
i a
i o
i •"?
o?
m
W
i H
p
o
02
g
3
I A
■	H
: p
!
i w
02
W
Ph
Ph
A
02
<1
H
A
Ph
:■ QD
M
i o
a
o
S
S
3
h
Hl
o
02
d
d
a
A
M
Q
£
M
02
02
g
8
• A
d I
JAS. P. HARRISON & CO., Printers.
ATLANTA, GEORGIA.
TERMS: $2.00 per Annum in Advance—Club Rates, 5 Copies for $8.00.
				

